# Exosomes as Theranostic Targets: Implications for the Clinical Prognosis of Aggressive Cancers

**DOI:** 10.3389/fmolb.2022.890768

**Published:** 2022-06-23

**Authors:** Richa Gulati, Dhruva Nandi, Koustav Sarkar, P. Venkataraman, K. M. Ramkumar, Priya Ranjan, Rajiv Janardhanan

**Affiliations:** ^1^ Department of Medical Research, Faculty of Medicine and Health Sciences, SRM Institute of Science and Technology, Kattankulathur, India; ^2^ Department of Biotechnology, School of Bioengineering, SRM Institute of Science and Technology, Kattankulathur, India; ^3^ Bhubaneswar Institute of Technology, Rourkela, India

**Keywords:** exosomes, tumour etiopathology, cancer immuno-editing, theranostics (combined therapeutic and diagnostic technology), clinical prognosis

## Abstract

Exosomes are extracellular vesicles produced by various cell types and extensively distributed in physiological fluids. Because of their significant role in cancer progression, they have been a focal point for the novel cancer therapy approach. Exosomes are highly efficient at transporting proteins, RNAs, and small drugs into cancer cells for therapeutic purposes. In addition to their prominent role as potential biomarkers for transporting targeted information from their progenitor cells, exosomes have also emerged as a new avenue for developing more effective clinical diagnostics and therapeutic techniques, also known as exosome theranostics. Lipids, proteins, and nucleic acids transported by exosomes were investigated as potential biomarkers for cancer diagnosis, prognosis, and future cancer treatment targets. The unique mechanism of exosomes and their therapeutic as well as diagnostic uses, also known as theranostic applications of exosomes in malignancies, are discussed in this review.

## Introduction

Cancer emerged as a severe hazard to humankind, with rising death rates worldwide (Zhao et al., 2019a). The tumor microenvironment (TME) plays a crucial role in cancer start and progression (Ragusa et al., 2017). Cell interaction is also vital in several diseases. To exist, reproduce, and spread, cancer cells require interaction with other health and immune cells (Wang et al., 2019a). Exosomes gained a lot of interest due to their cell-to-cell interaction property. The TME is altered by tumor-exosome interaction, promoting tumor progression, longevity, immune invasion, and evasion (Darband et al., 2018).

Exosomes, being the significant portion of TME, have shown to bear specific activities regarding tumor formation, angiogenesis, progression as well as drug resistance (Wang et al., 2019a). Exosome-related growth factors and cytokines may stimulate or inhibit lymphoid and immune cells of the TME, leading to immunodeficiency and the formation of tumors (De Visser et al., 2006; Yang et al., 2020a). Furthermore, exosomes have been successfully utilized in medication delivery (Zhao et al., 2020; H. Rashed et al., 2017). Also, exosomes’ drug carrier capacity (Srivastava et al., 2018a; Zhang et al., 2019) makes them ideal drug delivery vehicles. They were invented to give various medications, including tiny molecules, nucleic acids, and proteins, to animal models for cancer treatment. Exosomes are also secreted by a variety of cells which can be used as preclinical biomarkers in various cancers (Maji et al., 2017; He et al., 2018a; Farooqi et al., 2018; Das et al., 2019; Doyle and Wang, 2019; Mannavola et al., 2019; Yu et al., 2020). Alternative treatment techniques, such as inhibiting exosome synthesis and blocking exosome absorption to particular receptors, have also been considered potential cancer therapies (Qiu et al., 2020). Overall, the extensive applicability shows that several prospective therapeutic techniques involving the inhibition of tumor-derived exosome synthesis, release, or absorption are attractive avenues for the future development of cancer theranostics (Tai et al., 2018; Wang et al., 2020).

According to mounting evidence, exosomes can be released by various cells, including lymphocytes and fibroblasts. Exosomes generated from various cells have distinct properties (Cheng et al., 2020; Li et al., 2020). Cancer cell exosomes and MSC-derived exosomes are used to diagnose and treat various cancers, such as liver cancer, pancreatic cancer, etc., as demonstrated in murine experiments (Aqil et al., 2019; Nakamura et al., 2019; Qiu et al., 2020). Exosomes, irrespective of where they come from, can influence tumor development or suppression through various signaling channels (Pinheiro et al., 2018).

This review seeks to give an insight into exosome dynamics and progression in cancer theranostics, including their relationships, exosome uses, and exosome roles derived from different origins. The latest innovations in exosome technology will aid in developing cancer treatment applications shortly.

### Biogenesis of Exosomes

**FIGURE 1:**
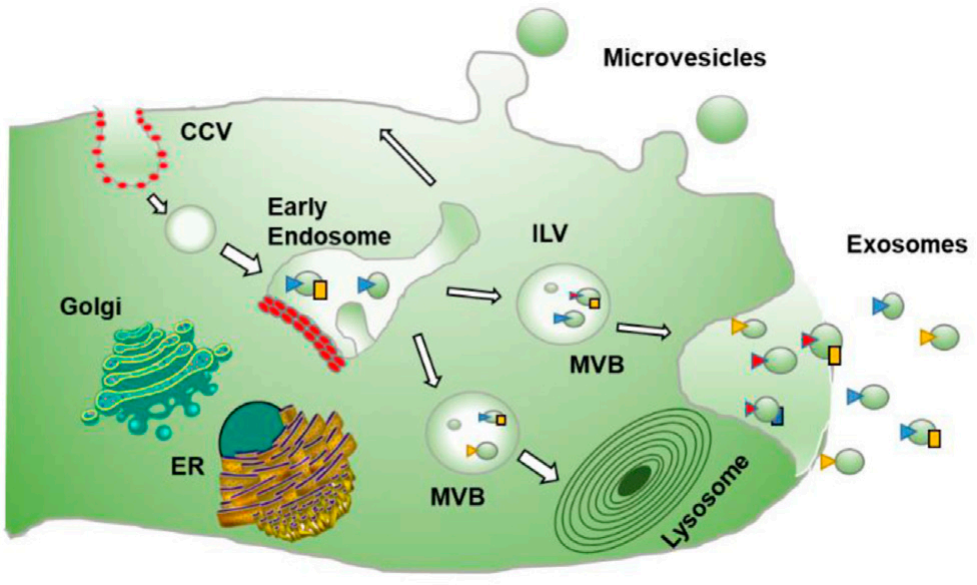
Process of formation of Exosomes: describes the formation of exosomes which is done by invagination of late endosomal membranes within large MVBs, resulting in the development of intraluminal vesicles (ILVs). Specific proteins are integrated into the invaginating membrane during this process, whereas cytosolic components are absorbed and confined inside the ILVs. When ILVs fuse with the plasma membrane, they are discharged into the extracellular region, known as “exosomes.” While microvesicles result from direct budding from the plasma membrane.

Extracellular Vesicles (EVs) are Nano-scale membrane vesicles that are actively released by cells. They occur through the outward budding of the plasma membrane or microvesicle pathway or inward budding by the inner body membrane or exosomal pathway. The vesicle formed through outward sprouting of the plasma membrane is called Multivesicular bodies (MVBs). Exosomes are vesicles of endocytic origin. The early endosome is formed by the intrusion of the plasma membrane inwardly, and the limiting layer of the subsequent endosome grows further to produce tiny vesicles, resulting in the development of MVBs. The MVB is distinguished by the formation of Intraluminal Vesicles following invagination of the inner body membrane (ILVs). During their development, cytoplasmic inclusions, transmembrane, and peripheral proteins are incorporated into ILVs. ILVs collected in the MVB lumen have two possible outcomes: a) fusion with lysosomes, causing the contents of the vesicles to break down [Multivesicular Endosomes or Bodies (MVBs or MVEs)], or b) fusion with the plasma membrane, releasing ILVs into the extracellular space as exosomes. On the other hand, direct budding from the plasma membrane forms microvesicles, which sequester sections of the cytoplasm (Figure 1). Microvesicle production is calcium-dependent and related to membrane asymmetry loss and cytoskeleton instability.

**FIGURE 2:**
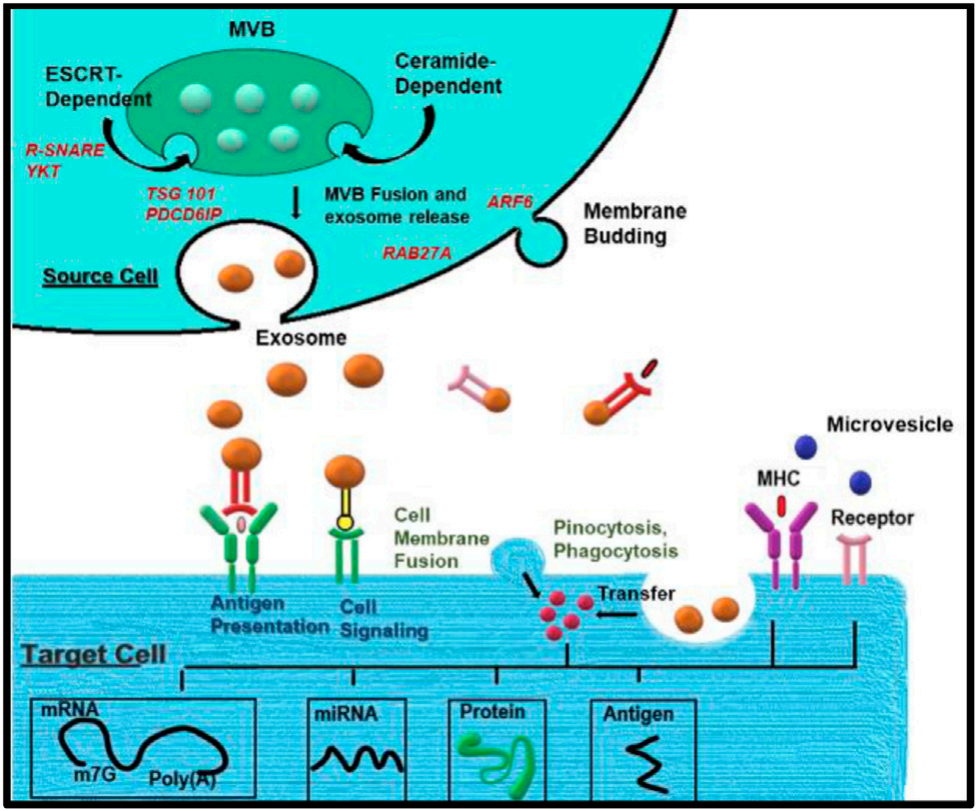
Biogenesis of exosomes: Exosome biogenesis, cargo, and secretion are depicted schematically. Exosomes are generated when the endocytic membrane is invaginated and ILV is created inside the cell. The payloads (RNAs, proteins, and lipids) are integrated into ILV *via* ESCRT-dependent or ESCRT-independent pathways during maturation, and MVBs are formed by the maturation of early endosomes. MVBs can be sent to lysosomes for breakdown or migrate along microtubules to fuse with the plasma membrane and release exosomes into the extracellular environment. MVB fusion with the cellular membrane is a delicate process that necessitates the presence of numerous key components such as Rab GTPases and SNARE complexes. Endocytosis, direct membrane fusion, and receptor-ligand interaction can all be used to transfer exosomal payloads from a source cell to target cells.

More specifically, the role of various proteins varies in different processes and origins of the exosomes. Leading cargo into ILV involves the ESCRT and other related proteins. ESCRRT includes PDCD6IP (also known as ALIX), TSG101, HRS, CD9, and CD82, etc., Other proteins include PLG2, DGKα, etc., After MVBs fuse with the cell membrane, exosomes are secreted. This process relies on small GTPases such as RAB27A and RAB27B. RAB7, RAB11, RAB31, and RAB35 in some cells or SNARES family proteins like YKT6, VAMP7, etc., Microvesicles (MVs) represent a relatively heterogeneous population of vesicles formed on the outward germination fission of cell membrane lipid microdomains and regulatory proteins such as ADP ribosylation factor 6 (ARF6). MVs participate in immune modulation by assisting in antigen presentation and transmitting MHC molecules and antigens (Figure 2). It is also possible to directly activate cell surface receptors through proteins and biologically active lipid ligands, transfer cell surface receptors, transcriptional factors, oncogenes, etc., Various RNAs, including mRNA, and miRNA, are contained in extracellular vesicles (EVs) and are functionally delivered to recipient cells.

## Exosome and Cancer Progression

Exosomes are produced in greater quantities (approximately 10 times) by tumor cells than normal cells, resulting in higher exosome concentrations in the blood of cancer patients (Liu et al., 2018). Due to paracrine subversion of microenvironments, Tumour-derived Exosomes (TEXs) regulate TME structuring, immune escape, and many more (Datta et al., 2018). As TEXs interact with various cells and deliver a large number of biomolecules, including oncogenic features, these organelles play a significant role in multiple malignancies through induction of angiogenesis, tumor metastasis, and aberrant metabolism, tumor progression, immune dysfunction, and drug resistance.

### Exosome- Angiogenesis and Tumour Metastasis

Neovascularization is a well-known biochemical phase in the progression of tumors and metastasis. Both stromal and tumor cells control tumor angiogenesis by producing numerous signaling molecules and proteins (Kerbel, 2008; Weis and Cheresh, 2011). Proangiogenic factors discovered in TEXs include angiogenin, TGF and many more (Webber et al., 2010; Kucharzewska et al., 2013; Wang et al., 2016).

Exosomal VEGF regulates angiogenesis through several pathways (Bryan et al., 2010; Weddell et al., 2018; Yang et al., 2020b). Mesenchymal Stem Cells derived exosomes increased VEGF expression in tumor cells by ERK1/2 and p38 MAPK pathways activation, which led to increased tumor development and angiogenesis (Zhu et al., 2012). MMP-2 and MMP-9 break down matrix proteins to structure the basement membrane of vessels and detach pericytes, while PDGF increases pericyte recruitment in the new tube (Guo et al., 2003; Webb et al., 2017).

Tumor metastasis begins with tumor cells splitting from the primary neoplasm and traveling to a distant organ location, which entails complicated biological processes. Exosomes promote metastasis by interfering with various cellular pathways (Jafari et al., 2021). Exosomes containing HSP90 have been shown to aid cancer cell migration and invasion by boosting the degradation of plasminogen and E-cadherin (Mccready et al., 2010).

Exosomes derived from metastatic breast cancer cells drive signaling pathways for PI3K/AKT mitogen-activated protein kinase (MAPK), which stimulate migration and invasion of cancer cells by degrading ECM components (Hendrix et al., 2010). Moreover, cancer invasion simultaneously requires invadopodia biogenesis and exosome secretion (Hoshino et al., 2013). Rab27a-dependent exosome secretion, in conjunction with cytokines and metalloproteinases, is thought to cause neutrophil aggregation, which contributes to the development of metastatic breast carcinoma (Bobrie et al., 2012).

According to several studies, exosomes, *via* modulating Wnt/β-catenin and ERK signaling pathways promote EMT (Hu et al., 2019). Exosomal miR-21 also reduces apoptosis in GC cells (Zheng et al., 2017) while also downregulating the PTEN tumor suppressor gene (Han et al., 2012). In BC cells, exosome IL-6 promotes tumor development (Yu et al., 2019), exosome HSP70 promotes tumor progression in cells of MSC (Li et al., 2016), and exosome TGF- promotes tumor growth in cells of LAMA84 (Raimondo et al., 2015). Exosomes miR-222 help stimulate the signaling pathway of NF-B and cause cancer cell motility and invasion (Ding et al., 2018).

### Exosomes and Cancer Immuno-Editing

The ability of the tumor to suppress the host immune system against cancer is the primary factor responsible for hampering immune surveillance. Additionally, TEXs mediate the communication between cancer and TME cells, which is critical for developing an immune-suppressive pro-tumor microenvironment. They limit the activation and function of helper and cytotoxic T cells, activating Tregs, inhibiting the cytotoxicity of NK cells, and decreasing leukocyte adhesion. Ye et al. (2014); Moloudizargari et al. (2018).

The PTEN/PI3K signaling pathway is activated by exosomes containing miR-301a-3p, leading macrophages to polarize from M1 to M2. By producing VEGF, TGF-b, and other soluble chemicals, M2 macrophages are important in angiogenesis, immunological suppression, tumor formation, and metastasis. In studies, it was observed that exosomes containing PD-L1 inhibited IFN production and activated apoptosis in T-cells. Exosomes can travel further and have a lower influence on immune cell activation than exosomes without PD-L1 (Kim et al., 2019). As a result, TEXs facilitate immune evasion and, as a result, tumor development *via* the PD-L1/PD-1 pathway Wang et al. (2018); Babajani et al. (2020).

Since the discovery of the Warburg effect, many attempts have been made to investigate metabolic reprogramming in cancer cells from oncogene acquisition to metastatic growth and treatment response. Malignancies have been shown to contain mutations, deletions, and variations in the number of copies of mitochondrial DNA (mtDNA), notably in response to therapy (Cormio et al., 2015; Kong et al., 2015; Dang et al., 2016). These changes might be unintended consequences of treatment-induced cancer cell selection. A recent study has shown that mitochondria and mtDNA might migrate across mammalian cells via dynamic intercellular organelle highways or nanotubes, proving that they are not exclusively located and propagated in somatic cells of higher species (Pasquier et al., 2013; Jackson et al., 2016).

### Exosomes and Their Role in Cancer Metabolism

Alterations in cell metabolism are one of the hallmarks of cancer. Exosomes have long been thought to be essential mediators in cancer progression, regulating extracellular communication with cancer cells (Barros et al., 2018). It is well-established that Tumour Microenvironment (TME) development is essential for cancer progression. Exosome-mediated metabolic reprogramming is observed in cancer cells and stromal cells in the TME, implying that exosome-mediated metabolic reprogramming plays a significant role in the progression of aggressive cancers (Yang et al., 2020c). The cancer cells affect stromal cell metabolic remodeling, which operates as a feedback loop to help cancer cells grow faster (Tan et al., 2020).

Cancer-Associated Fibroblasts (CAFs), a bulk of TME cell types, are characterized by the conversion to a synthetic phenotype which could be ascertained by the expression of markers such as α-smooth muscle actin (α-SMA), fibroblast-specific protein-1 (FSP1/S100A4), and fibroblast activation protein (FAP) (Östman and Augsten, 2009). Cancer development can cause normal fibroblasts to act as a precursor of CAFs. CAFs promote tumor growth and progression by inflammatory and growth factor production, among other mechanisms (Xing et al., 2010).

The Warburg effect is defined as cancer cells’ reliance on aerobic glycolysis even in normoxia, a frequent feature of many cancer types. This causes glucose to be diverted to lactate, resulting in low pH circumstances that affect TME (Warburg et al., 1927; Salimian Rizi et al., 2015). CAF glycolysis can be aided by cancer cells. CAFs can then feed cancer cells with metabolites and aid cell growth *via* the TCA cycle and OXPHOS. This phenomenon is known as the “Reverse Warburg Effect” (Martinez-Outschoorn et al., 2011). Lactate export in CAFs and lactate uptake by cancer cells has recently been proven to aid the turnover rates in cancer metabolism (Diehl et al., 2018). MCT1 and MCT4 are lactate symporters that play a crucial role in building a lactate shuttle system. MCT4 promotes lactate export, whereas MCT1 promotes cellular lactate uptake. This lactate is utilized as fuel by various cancer cells under complete aerobic conditions, as it can be converted into glucose in the liver by the Cori cycle. Caveolin-1 (Cav-1, a membrane-bound scaffolding protein involved in endocytosis, signaling, cell motility, and cholesterol distribution) may also contribute to cancer progression by controlling the metabolism of CAFs, according to more recent research (Nwosu et al., 2016).

Incremental evidence suggests an explicit role of exosomes in metabolic reprogramming between CAFs and cancer cells. Cancer cells cultivated with exosomes have been shown to have lower OXPHOS and increased glycolysis, as evidenced by increased glucose absorption and lactate production (Zhao et al., 2016). CAF-derived Exosomes (CEDs) induce a hypoxia-like environment in cancer cells, resulting in an increase in glutamine reductive carboxylation, which is a crucial mechanism for tumor cells to develop low-oxygen environments. Cancer cells rely on this reductive glutamine carboxylation when stromal exosomes are present. According to a study, exosomes from pancreatic and prostate CAFs were shown to include intact intracellular metabolite pool components such as amino acids, acetate, stearate, palmitate, and lactate. Exosomes can fuel the TCA cycle in a similar way as macropinocytosis in prostate cancer, and the metabolite enrichment resulting from exosomes is independent of Kras mutation (Zhao et al., 2016). Furthermore, the critical involvement of exosomes produced by cancer cells in modifying fibroblast metabolism and increasing glycolysis was established in breast cancer cells (Yan et al., 2018; Sung et al., 2020). Exosomes produced from cancer cells may also trigger the expression of MCT4 in CAFs, allowing the cancer cells to export β-HB and lactate, and cancer cells expressing MCT1 use lactate to increase OXPHOS levels (Yan et al., 2018; Wu et al., 2020).

### Exosomes and Drug Resistance

Exosomes act as a barrier to drug permeation and impart drug resistance by transporting cargo from resistant cells to susceptible ones (Sansone et al., 2017). According to numerous studies, exosomes are thought to play a role in modulating chemosensitivity to recipient cells by delivering the resistant phenotype (Qin et al., 2017). The trafficking of ncRNAs such as miRNAs and lncRNAs by exosomes is a good approach for cancer cells to gain treatment resistance. In ovarian cancer, the exosomal transmission of miR-433, for example, can promote cellular senescence and hence increase paclitaxel resistance (Sousa et al., 2015).

The capacity of cancer cells to adapt to a stressful environment is one of their most essential characteristics (Weiner-Gorzel et al., 2015). Exosomes retain CSC self-renewal as property by carrying these cargos, increasing resistance to numerous cancer therapies (Luga et al., 2012; Gradilla et al., 2014; Ayob and Ramasamy, 2018).

By upregulating PI3K/Akt signaling *via* miR-21, exosomes from M2-macrophages decreased apoptosis and cisplatin resistance in GC cells (Chairoungdua et al., 2010). The lncRNA urothelial cancer associated-1 (UCA1) can increase tamoxifen resistance in estrogen receptor-positive MCF-7 cells *via* the mTOR signaling pathway (Fan et al., 2014). By activating the Wnt signaling pathway, exosomal transfer of the lncRNA UCA1 can increase bladder cancer cell chemoresistance (Liao et al., 2016). Exosomes derived from MSCs helped transmit the lncRNA PSMA3-AS1, which provides resistance to proteasome inhibitors (Xu et al., 2016).

Another method of acquired drug resistance in cancer is proto-oncogene transmission via exosomes, which activates the PI3K/Akt signaling pathway. Anti-apoptotic signaling cascades are stimulated by the penetration of proteins such as Ras, Src, and MAPK families into recipient cells, resulting in a rise in drug-resistant cells (Xu et al., 2019). Exosome-mediated drug transporter molecules have been linked to the spread of treatment resistance in various cancer types (Dutta et al., 2015). Exosome trans locates chloride channel one enhanced GC cell line SGC-7901, conferring vincristine resistance (Sinha et al., 2021).

## Clinical Application of Exosomes

Exosomes have emerged as a new avenue in developing more effective clinical diagnostics and therapeutic techniques.

### Exosome as Biomarkers

Exosomes are suitable biomarkers for cancer diagnosis in a non-invasive manner. For example, Exosomal caveolin-1 and CD63 are the non-invasive markers for melanoma (Zhao et al., 2019b). In numerous cancer types, exosomal lncRNA was linked to tumor classification (III/IV), tumor stage, and lymph node/distant metastases, either in combination with miR-21 or on its own (Peinado et al., 2012). Within TEXs, pancreatic ductal adenocarcinoma cells produce a type II transmembrane protein called cytoskeleton-associated protein 4 (CKAP4). CKAP4 can be employed as a biomarker for pancreatic ductal adenocarcinoma diagnosis, staging, and targeted treatment, according to a study of patient blood samples (He et al., 2018b). Exosomes extracted from glioblastoma patients’ plasma also included TrkB (Tropomyosin receptor kinase B) expression, suggesting that this receptor might be employed as a biomarker for glioblastoma diagnosis (Kimura et al., 2019).

Exosomes have recently been discovered to have DNA and RNA sources in circulation (Pinet et al., 2016). Exosome DNA (exoDNA) was significantly predictive of the outcome of cancer therapy and progression in patients with metastatic malignancy (San Lucas et al., 2016). MicroRNAs, in addition to DNA, are valuable indicators of cancer diagnosis and progression. MiR-21 and miR-155, for example, have been reported to be considerably elevated in recurrent tumors compared to original tumors in lung cancer (Sohn et al., 2015; Bernard et al., 2019).

Exosomes can also be utilized to track how well a patient responds to treatment. In NSCLC patients and melanoma treated with an antibody’s combination of PD1/PD-L1, there was an essential link between exosomal PD-L1 mRNA expression levels and therapeutic response. Exosomes derived from plasma exhibited greater PD-L1 mRNA levels, indicating a stronger reaction (Hornick et al., 2015).

### Exosomes in Immunotherapy

Despite clinical advancements, cancer remains the leading cause of death globally. Researchers are also working on a new technique to use exosomes to modify immune responses against cancer: cell-free vaccinations. Exosomes produced from DCs are loaded with a tumor-specific antigen in a classic strategy to re-establish anticancer immunity; miRNAs or exosomes alone are used to induce an immune response in the recipients. After a tumor peptide pulse, André et al. isolated exosomes from DCs and gave them to mice with preexisting tumors, resulting in tumor rejection mediated by T-cell activation (Munoz et al., 2013). Exosomes expressing modified IL-2 were later discovered to be capable of causing a considerable regression of a preexisting tumor by targeting the antigen-specific Th1-polarized immune response and cytotoxic T lymphocytes (CTL) with exosomes expressing modified IL-2 (André et al., 2004). Because DCs have a lot of lactadherin on their surfaces, which enhances exosome absorption, exosomes produced from DCs are beneficial in treatment (del Re et al., 2018). The functional moieties of exosomes contribute in the induction of antitumor immune responses, both innate and adaptive (Yang et al., 2007).

IFN and polycytidylic acid were used as adjuvants to create mature exosomes formed from DC with increased capacity for Th1 cell activation (Viaud et al., 2011; Syn et al., 2017). MS-275, a histone deacetylase inhibitor, and Hsp70 release increased lymphocyte proliferation and NK cytotoxicity (Pitt et al., 2016). Exosomes formed from heat shock tumors were discovered to have higher immune-stimulating capabilities due to increased MHC expression and cytokine quantities (Xiao et al., 2013). After being pre-incubated with HSP70 surface-positive exosomes, NK cells promote apoptosis in colon cancer cells by releasing granzyme B (Chen et al., 2006). Extracellular HSP70 was later discovered to activate macrophages, and this immunological modulator effect depended on HSP70’s capacity to translocate into the plasma membrane from the cell surface (Gastpar et al., 2005).

### Exosomes and Delivery of Drugs

**FIGURE 3:**
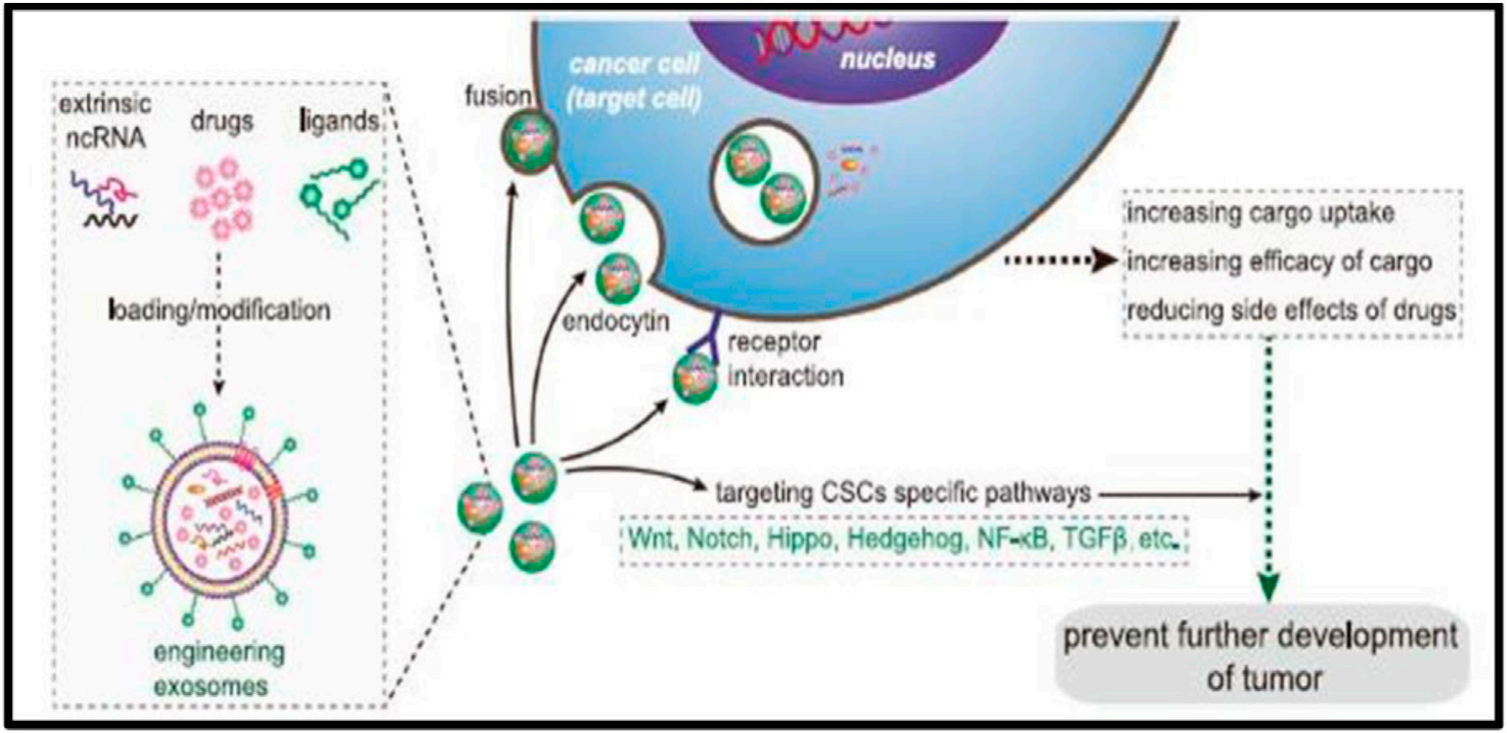
Precisely targeting the tumor with engineering exosomes as a delivery carrier. Exosomes are carriers with natural delivery ability, which have the characteristics of precisely targeting and high bioavailability. After being loaded into exosomes, anticancer drugs and/or extrinsic ncRNA can directly target cancer cells or CSCs specific pathways and prevent the further development of tumors. Additionally, the surfaces of exosomes can also be modified with the ligands corresponding to receptors overexpression on cancer cell surfaces, which improves the cellular uptake efficiency of exosomes by cancer cells. ncRNA noncoding RNA, CSCs cancer stem cells.

Exosomes are non-toxic, non-immunogenic, and have a greater penetration rate through tumor cells than conventional drug delivery methods. Exosomes, due to their stable load capacity, can carry proteins, miRNAs, small interfering RNAs (siRNAs), and other therapeutic compounds (Figure 3) (Vega et al., 2008). Exosomes also have the advantage of crossing biological barriers such as the blood-brain barrier (BBB) and settling in specific areas (Brannon-Peppas and Blanchette, 2004; Hood et al., 2011; Zhao et al., 2018).

The modified exosomes have significant anti-neoplastic effects in pancreatic adenocarcinoma cells of humans (Tominaga et al., 2015). Exosomes carrying paclitaxel derived from human prostate adenocarcinoma cells, which are androgen-sensitive, were found to negatively affect the cancer cells’ viability (Pascucci et al., 2014). In BC cells, exosomes expressing anticancer action was demonstrated when cholesterol-modified miRNA 159 and doxorubicin were administered along with metalloproteinase 15 (ADAM15) (A15-Exo) and disintegrin (Saari et al., 2015).

Exosomes might be used to deliver anticancer proteins to cancer cells with pinpoint accuracy. TNF-related apoptosis-inducing ligand (TRAIL) activates death receptors in cancer cells, causing them to perish Gong et al. (2019). Tumor cells lacking LATS1/2, a Hippo pathway kinase, have produced exosomes rich in nucleic acid boosting tumor immunogenic behavior (Rivoltini et al., 2016).

Because clinical use of nucleic acid medications is limited due to insufficient delivery methods, exosomes may be a good vehicle for delivering therapeutic nucleic acids. RNAs (siRNA and miRNA) have been delivered to tumor cells using exosomes. Exosomes derived from adipose stem cells were utilized to provide miR-122, making hepatocellular carcinoma chemoresistant (Lou et al., 2015; Moroishi et al., 2016). Exosomes were also utilized to transport siRNA against RAD51, which reduced RAD51 expression in cervical and fibrosarcoma cancer cells, resulting in significant cell death in the recipients (Zhang et al., 2020).

Clinically, nanotechnology-based drug delivery systems are among the most promising tools to achieve this goal. Compared with liposome nanomaterials, metal nanomaterials, and polymer nanomaterials, exosomes as carriers can overcome the shortcomings of poor bioavailability and reduce non-targeted cytotoxicity and immunogenicity (Sun et al., 2010; Srivastava et al., 2018b). And exosomes contain transmembrane and membrane anchoring proteins, which enhance endocytosis and thus promote the transfer of their contents (Blanco et al., 2015). For instance, Kim et al. found that paclitaxel-loaded macrophage-derived exosomes significantly increased cell uptake in 3LL-M227 mouse Lewis lung cancer cell line compared to paclitaxel-loaded liposomes (Kamerkar et al., 2017; Luan et al., 2017).

Scientists developed a new technique for decreasing drug resistance traits using vesicles derived from tumor cells packed with antitumor drugs. Chemotherapeutic medications were bundled into tumor cell-derived vesicles to improve medication access into the nucleus. TRCs preferentially absorbed these vesicles, allowing antitumor medications to be released and assisting in reversing treatment resistance in TRCs *in vitro* (Shtam et al., 2013). According to research, curcumin stimulates myeloid cells through exosomes, resulting in anti-inflammatory effects and monocyte death (Ma et al., 2016). Moreover, compared with free drugs, exosome-based delivery platforms can significantly reduce side effects. The use of engineered exosomes containing miR-21 sponge constructs could downregulate the expression of miR-21 in glioma cell lines U87-MG and C6, thereby upregulating the target genes PDCD4 and RECK of miR-21 and preventing their malignant behavior (Kim et al., 2016).

Recent studies have shown that exosome surface modification is performed using oligonucleotide binding methods. Such cargo may potentially alter cell function and alter cell-to-cell transport (Monfared et al., 2019). Triple-negative breast cancer is one subtype of breast cancer with the most metastatic and recurrent characteristics. A study by Li et al. (Yerneni et al., 2019) modified the surface of the exosomes with a peptide targeting the mesenchymal-epithelial transition factor gene (c-Met), for hepatocyte growth factor, which is overexpressed on triple-negative breast cancer cell surfaces Li et al. (2018).

Since exosomes can also affect CSCs by targeting CSC-specific signaling pathways, such as Wnt, Notch, Hippo, Hedgehog, NF-κB, and TGF-β pathways, selective targeting of CSCs via the above pathway using exosome loading inhibitors (miRNA or siRNA) is considered to be achievable. Existing results have shown that exosomal Wnt from fibroblasts could induce dedifferentiation of cancer cells to promote chemotherapy resistance in CRC, suggesting that interference with exosomal Wnt signaling could help improve chemosensitivity and treatment window. Exosomes are projected to be beneficial in treating cancer as a whole, with uses in biomarker detection, tumor immunology, and drug delivery. They might potentially alter medicine as a significant participant in theranostic oncology.

### Antitumour Vaccine Using Exocrine System

TEXs have a dual effect on the immune system, i.e., immunosuppressive or immunostimulatory effects. Numerous research has shown that TEXs can interfere with the maturation of DCs, weaken the activation of NK cells, induce suppressor cells of myeloid origin, and transform macrophages into tumor-promoting phenotypes (Raghav et al., 2012; Whiteside, 2016; Chen et al., 2017). The activated CD8^+^ effector T cells in the circulation system of cancer patients were induced apoptosis by TEXs, which was one of many immunoinhibitory mechanisms of TEXs and suppressed the patient’s general immune system. As a carrier for delivery products, exosomes can initiate antitumor immune responses with significant therapeutic effects on tumor progression (Olejarz et al., 2020). In a mouse model with melanoma, mice were treated with α-galactosylceramide/ovalbumin-loaded exosomes, which induced an early T cell response and eventually slowed tumor growth compared to the control group. Abundant alpha-fetoprotein in exosomes produced by *in vitro* cultured HCC could stimulate the antigen-presenting function of DCs, stimulate the proliferation of CD8^+^ T cells, regulate the secretion of inflammatory cytokines (reducing IL-10 and TGF-β secretion and increase the secretion of IFN-γ and IL-2), and enhance immune-induced apoptosis (Rao et al., 2016; Huang et al., 2018; Wang et al., 2019b). According to Xie et al. (2010), a vaccine developed by exosomes was effective in antitumor immunity. Their study used exosomes from MM (multiple myeloma) cells to stimulate antitumor immune responses and generate prophylactic immunity in MM cell lines (Shi et al., 2018; Li et al., 2019). TEXs recovered and enriched from patient sera may provide an optimized, individual-specific antigen source for DCs vaccination (Xie et al., 2010). How to make full use of the advantages of TEXs, and bypass their disadvantages to regulate tumor immunity needs further research, which has great potential in the application of cancer targeted therapy (Gu et al., 2015; Liu et al., 2015).

## Conclusion and Future Prospects

We believe that the intricate nuances associated with the biogenesis as well as the prospecting of exosomes as a theranostic target for aggressive cancers is yet to be exploited to its fruition as cumulative evidence from populations with heterogeneous genetic makeup are yet to be documented. Systematic studies concentrating on evaluating the theranostic potential of exosomes are being conducted in the western hemisphere equipped with a well-nuanced healthcare ecosystem and in resource-limited healthcare ecosystems prevalent in Low-And-Middle-Income Countries (LMICs) as India. Exosomes could have prospected as plausible biomarkers of aggressive cancers as they play an intrinsic role in the etiopathogenesis as well as clinical prognosis. Based upon the origin of the exosomes, we hypothesize that it could be prospected as an attractive theranostic target to create novel immune-surveillance strategies to enable large-scale screening of aggressive cancers at the community level, taking into account the wide variation in the host genetics along with socio-cultural norms. This will help predict the emergent cancer hotspots, which could be seamlessly visualized on digital platforms such as Google Earth. Such a strategy will enable administrators/policy decision-makers at local, regional, national, and global levels to seamlessly allocate the clinical resources to mitigate the lurking epidemic of various cancer at the community level. This kind of community-driven strategy for cancer screening would form the rationale for developing comprehensive cancer management/mitigation strategies which will use computational platforms to develop fruganomic, cutting-edge, niche-specific cancer control strategies intrinsic to the health status of the populace subsisting in a particular area.
